# Perioperative 3D transoesophageal echocardiography. Part 2: clinical applications

**DOI:** 10.1016/j.bjae.2024.04.008

**Published:** 2024-06-17

**Authors:** L. Sharifi, C. Luzzi, A. Vegas

**Affiliations:** Toronto General Hospital, University of Toronto, Toronto, ON, Canada

**Keywords:** anaesthesia, cardiac procedures, echocardiography, three-dimensional, thoracic surgery

**Learning objectives**

After reading this article, you should be able to:

• List the main applications of three-dimensional transoesophageal echocardiography (3D-TOE) in cardiac surgery and interventional cardiology across valvular disease, chamber quantification, cardiac masses and percutaneous procedures.
• Identify the advantages and disadvantages of the technology compared with 2D-TOE and other imaging modalities.
• Discuss the uses of 3D models created using 3D-TOE in education and procedural planning.

**Key points**

• Three-dimensional transoesophageal echocardiography (3D-TOE) has become increasingly adopted into perioperative practice.
• It displays structures as rendered models with or without 3D colour Doppler information.
• 3D-TOE allows custom 2D planes to be visualised in any orientation.
• Custom models allow new data to inform procedural planning.
• Technology remains limited by complexity and spatiotemporal resolution.

Three-dimensional transoesophageal echocardiography (3D-TOE) has advanced significantly since its first clinically usable implementation in 2008. This article considers its current clinical applications and follows part 1, which discusses the underlying technology behind 3D-TOE.^1^ Modern workflows allow rapid acquisition, processing and real-time display of a 3D dataset, facilitating perioperative decision-making. Three-dimensional transoesophageal echocardiography complements 2D-TOE by better visualising individual patient anatomy and pathology and can be superior to 2D-TOE in assessing valvular pathology, ventricular function, cardiac masses and congenital heart lesions (Table 1).^2^ It is indispensable for real-time guidance during percutaneous interventions for structural heart disease. Just as M-Mode echocardiography is still in daily use, 3D-TOE does not replace its 2D or spectral Doppler counterparts. As with all imaging, the technology has strengths and limitations which an experienced operator will appreciate, blending different echocardiographic and other modalities together to support their clinical reasoning.

**Table 1:** Areas in which 3D-TOE offers advantages over comparable 2D-TOE imaging. ASD, atrial septal defect; AVA, aortic valve area; EF, ejection fraction; LAA, left atrial appendage; LVOT, left ventricular outflow tract; MPR, multiplane reconstruction; MV, mitral valve; MVA, mitral valve area; PISA, Proximal isovolumetric surface area; RWMA, regional wall motion abnormalities; TAVI, transvalvular aortic valve insertion; TV, tricuspid valve; VCA, vena contracta area.

Structure and pathology	Applications where 3D-TOE can provide an advantage
**Mitral valve**	
*Mitral regurgitation*	Mechanism, models, VCA, 3D-PISA
*Mitral stenosis*	MPR planimetry MVA
**Aortic root**	
*Aortic regurgitation*	Mechanism, models, VCA, dimensions, cusp measurements
*Aortic stenosis*	LVOT area, AVA by planimetry
**Tricuspid valve**	
Identify specific leaflets, annulus measurements	
**Ventricles**	
*Left ventricle*	Volumes, EF, strain, RWMA
*Right ventricle*	Volumes, EF
**Cardiac masses**	
Location, attachment, size	
**Congenital heart disease**	
3D Model printing of complex heart lesions, heightened understanding of complex anatomy before and after the procedure	
**Interventional structural heart**	
Trans-septal puncture, ASD closure, MV interventions, TV interventions, LAA occluder placement, TAVI	
**3D Model printing**	
Complex heart lesions, educational tools	

Although still limited by spatiotemporal resolution, current technology offers real-time imaging across all 3D echocardiographic modes. It can integrate colour flow Doppler (CFD) with rendered 3D volumes in real time at usable frame rates with a single heartbeat capture, minimising artefact. Multi-planar reconstruction (MPR) aligns custom 2D planes anywhere within a 3D dataset, enabling precise measurements not possible with 2D-TOE. Semi-automated analytic software on ultrasound machines can model valves and ventricles, aiding detailed assessment and procedural planning.

Recent American Society of Echocardiography (ASE) guidelines for both surgical decision-making in the operating theatre and intervention for structural heart disease expect all perioperative echocardiographers to be proficient in 3D-TOE.^3^^,^^4^ However, the ASE does however acknowledge that the complexity of acquisition and analysis can challenge the incorporation of 3D-TOE into a busy perioperative workflow.^5^

## Valvular disease

Transoesophageal echocardiography is an excellent tool for interrogating native and prosthetic heart valves. Peri-procedural imaging can confirm and refine anticipated findings, identify unexpected pathology and assess post-procedural results.^3^, ^4^, ^5^ Two-dimensional transoesophageal echocardiography uses predefined imaging planes to slice through valves, requiring the echocardiographer to reconstruct mentally the 3D anatomy. This limits the appreciation of dynamic valvular function and makes it problematic to illustrate findings to surgeons. Three-dimensional transoesophageal echocardiography can show the entire valve in a single display, with user manipulation enabling viewing from any perspective (Table 2). Cropping can slice the 3D dataset like a virtual anatomic dissection, demonstrating structures of interest.

**Table 2:**
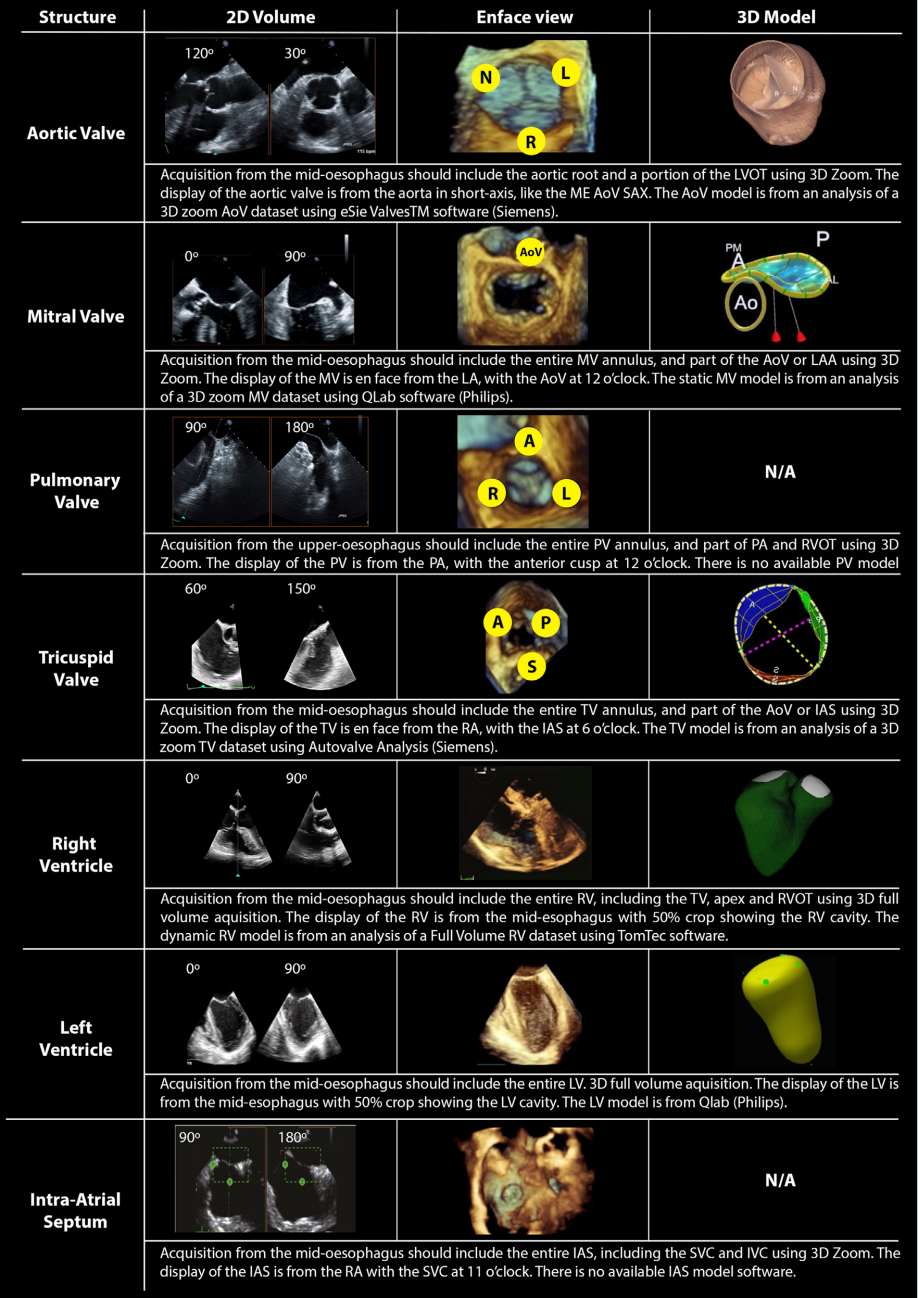
Summary of the acquisition and standardised presentation of 3D-TOE datasets. Each structure listed is first visualised and optimised using biplane imaging, as shown. The 3D dataset is acquired and rendered before being cropped and oriented in a standardised way, as shown using live or saved data. For some structures, a 3D parametric model may provide additional information (see main text). A, anterior leaflet; AL, anterolateral; Ao, aortic root; IVC, inferior vena cava; L, left cusp; ME AoV SAX, mid-oesophageal aortic valve short axis view; N, non-coronary cusp; P, posterior leaflet; PA, pulmonary artery; PM, posteriomedial; R, ; RA, right atrium; RVOT, right ventricular outflow tract; S, septal leaflet; SVC, superior vena cava.

### Mitral valve

**Fig 1:**
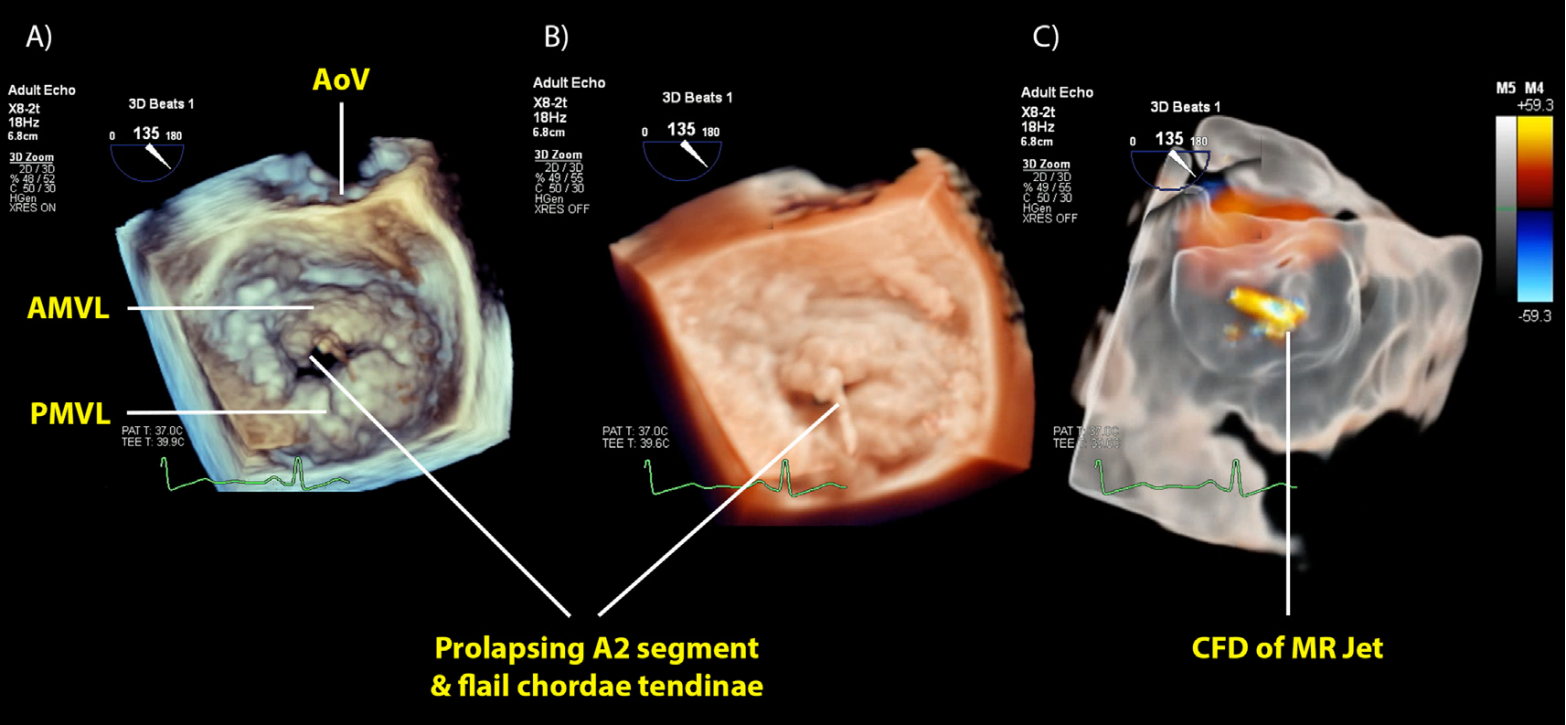
Three-dimensional transoesophageal echocardiography *en face* view of the MV. (A) Shows a pathologic MV with Barlow's disease, bileaflet prolapse, a coaptation gap, a flail of the A2 segment of the AMVL, and a ruptured chorda tendinae flicking in and out of the LA. The valve appears in mid systole from the standard surgical *en face* view from the LA perspective with the AoV in the 12 o'clock position. It uses a standard 3D render where brown shading is closer and blue shading is further from the viewer. (B) Shows part (A) but with tissue-like rendering and 3D ray tracing to simulate a light source. The ‘shadow’ generated by placement of the light source makes the ruptured chordae and prolapsed leaflet more easily discernible. (C) The tissue is translucent, so-called ‘glass’ rendering, aiding identification of the MR jet origin. AoV, aortic valve; AMVL, anterior mitral valve leaflet; CFD, colour flow Doppler; LA, left atrium; MR, mitral regurgitation; MV, mitral valve; PMVL, posterior mitral valve leaflet.

**Figure S1:**
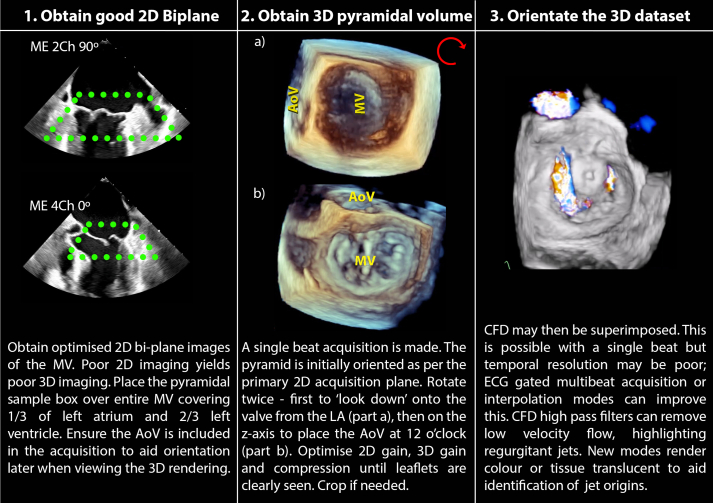
MV en face view. This summarises the directions to obtain the surgical en face view of the MV. Specific steps vary between different platforms but are broadly similar. Abbreviations: AoV, aortic valve; CFD, colour flow Doppler; LA, left atrium; MV, mitral valve.

Mitral valve (MV) imaging has become synonymous with 3D-TOE because it attains excellent images (Fig. 1, Fig. 1 online video). The valve is more than just its leaflets, and pathology affecting valve function may involve the annulus, chordae, left atrium (LA) and left ventricle (LV).^6^ Three-dimensional transoesophageal echocardiography can interrogate each component of the MV apparatus with superior diagnostic accuracy and localisation compared with 2D-TOE (Supplementary Fig. S1).^2^^,^^6^

Supplementary video related to this article can be found at https://doi.org/10.1016/j.bjae.2024.04.008

The following is/are the supplementary data related to this article:

Video S1 This video focuses on the mitral valve.2 Video S1 - mitral valve

**Figure S2:**
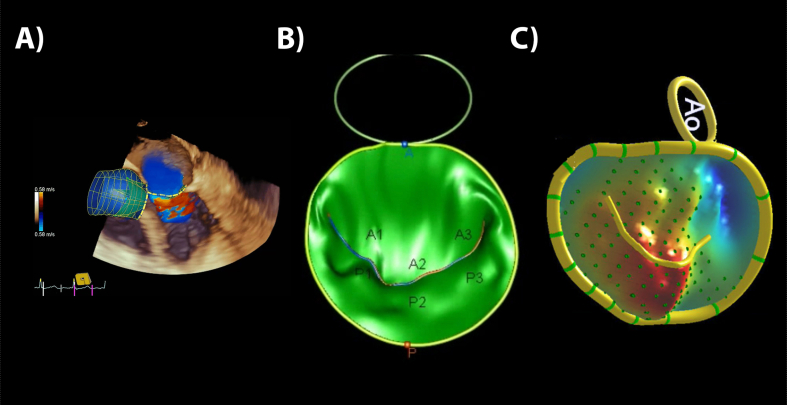
Parametric MV models. (A) This is a dynamic Siemens (Erlangen, Germany) model of the MV, which overlies the 3D full volume dataset and integrates CFD across the entire cardiac cycle. (B) This is a dynamic MV model during systole analysed using TomTec (Munich, Germany) software. (C) This shows a static mid-systole Philips (Amsterdam, Netherlands) MV model, identifying scallops on both leaflets and the coaptation line. Each software automatically derives and displays indices for these models. Abbreviations: CFD, colour flow Doppler; MV, mitral valve.

Strengths of 3D-TOE are the ability to rotate and examine the rendered 3D image from any perspective, use MPR to create a 2D view in any plane, or analyse structures using modelling software. The latter generates static or dynamic parametric models, providing details about valve dimensions and motion (Supplementary Fig. S2). These data can inform whether leaflet or chordal interventions are necessary in addition to an annuloplasty.

#### Mitral regurgitation

**Figure S3:**
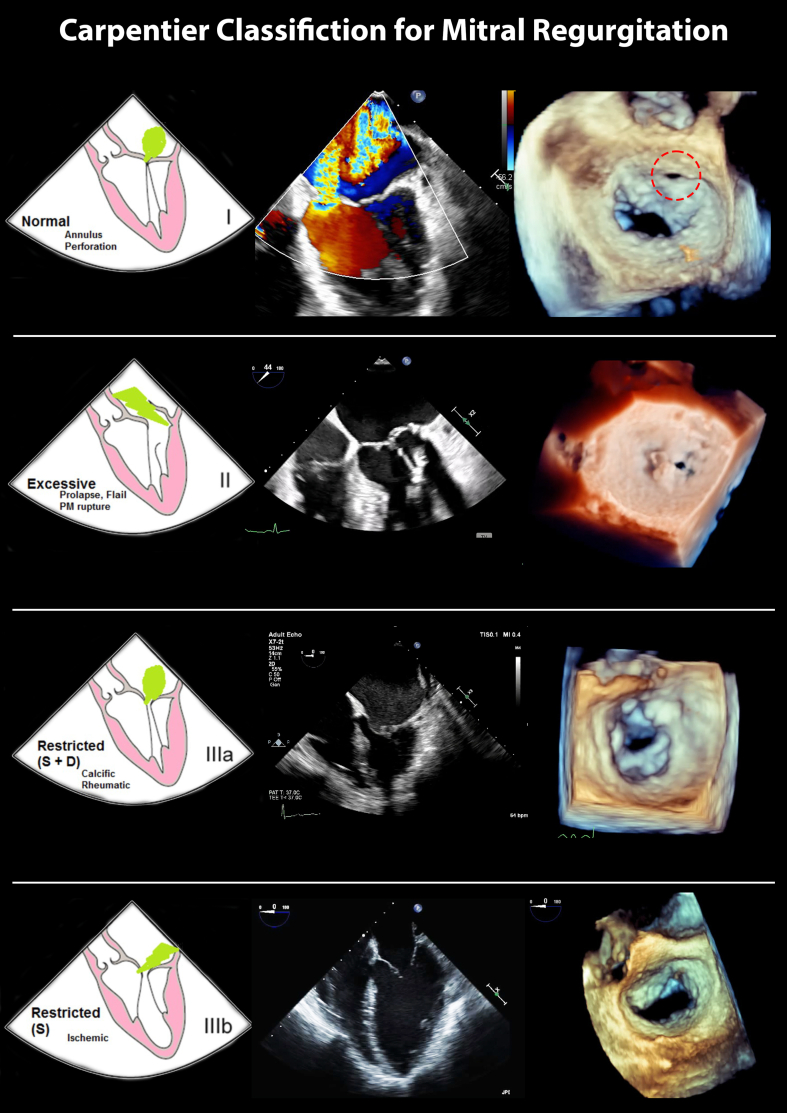
Carpentier classification of MR. Category I is MR with normal leaflet motion, typically from clefts or perforation. The example shows a colour jet that is not originating from the coaptation line. The 3D en-face view shows a perforation in the anterior MV leaflet near the annulus (red circle). Category II is MR with excessive leaflet motion. The example shows posterior leaflet prolapse in the 2D view, although it is unclear which segment is involved. The 3D en-face view highlights a flail P2 segment. Category IIIa is leaflet restriction in systole and diastole, often caused by calcific or rheumatic valves. The 2D image shows a thickened rheumatic valve, also seen in the 3D en-face view in diastole. Category IIIb is restriction in systole only from LV ischaemia, leading to annular dilatation or RWMA that tether the chordae tendinae. Abbreviations: MV, mitral valve; MR, mitral regurgitation; RWMA, regional wall motion abnormalities.

Surgical treatment of mitral regurgitation (MR) involves valve repair or replacement. Grading MR severity determines prior surgical planning and postoperative assessment of interventions. Understanding the mechanism of MR as described by the Carpentier classification (Supplementary Fig. S3) guides surgical options and improves the chance of successful valve repair.^2^ The inability to account for all mechanisms of valve dysfunction, such as prolapsing segments, clefts, or restricted motion from chordal tethering, contributes to repair failure. Two-dimensional transoesophageal echocardiography may miss or incorrectly localise pathology because of imaging plane misalignment. Three-dimensional transoesophageal echocardiography renders the entire MV in an *en face* view replicating the valve orientation seen when the surgeon looks through an open LA. Figure 1 and Figure 1 online video illustrate different options to display the MV using 3D-TOE.

Colour flow Doppler identifies regurgitant jets from leaflet coaptation defects or paravalvular leaks. The jet's origin, direction and components of flow convergence, vena contracta (VC) and jet area help to characterise regurgitation severity. It is often difficult to follow the origin and direction of eccentric and multiple jets using 2D-CFD. Three-dimensional colour flow Doppler overcomes this by presenting the entire MV and MR jet together, albeit at a lower spatiotemporal resolution. Rendering the tissue or the colour flow in the 3D dataset translucent may help to identify the origin of a regurgitant jet. Qualitative, semi-quantitative and quantitative methods can assess MR jets, each with limitations.^7^ Three-dimensional transoesophageal echocardiography improves accuracy compared with 2D-TOE, specifically for measuring VC area (VCA) and the flow convergence proximal isovelocity area (PISA).^7^

**Figure S4:**
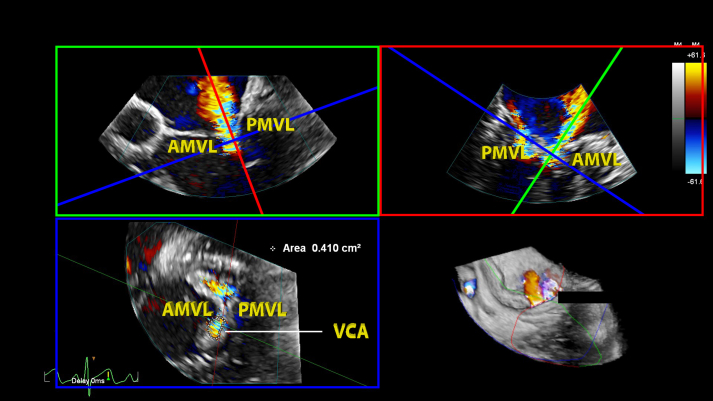
Measuring VCA using MPR. As described in the main text, VCA measurements can estimate MR severity. The 2D image in the green box derives from the 3D dataset and resembles a conventional 2D mid-oesophageal 4 chamber view of the MV. The red box is a 2D plane perpendicular to the green. Both planes align across the MR jet orifice. The blue plane aligns perpendicular to both the red and green planes at the level of the RO. The RO appears in short axis in the blue box and traced using planimetry to yield the VCA of 4.10 cm^2^ (see white dotted line in blue box). Note that in this example, there is a second MR jet seen in both the red and blue panels that is not being measured. 3D-TOE can help in isolation and measurement of multiple jets like this, which can be challenging with 2D imaging alone. Abbreviations: AMVL, anterior mitral valve leaflet; MPR, multiplane reconstruction; MR, mitral regurgitation; MV, mitral valve; PMVL, posterior mitral valve leaflet; RO, regurgitant orifice; VCA, vena contracta area.

Vena contracta is a simple semi-quantitative method for grading MR severity, which correlates with more complex quantitative measurements of regurgitant blood volume and regurgitant orifice (RO) area.^6^^,^^7^ The VC is the smallest, highest velocity region of jet flow sited at or near the RO at mid-systole. Assuming a circular defect, the VC width reflects the RO diameter, with greater width signifying more severe MR. However, rather than circular, 3D-TOE shows the RO is often elliptical or irregular, leading to misleading 2D VC measurements.^6^ Using MPR with a 3D-CFD dataset, one can position a 2D plane exactly at the RO level before superimposing CFD flow (Supplementary Fig. S4). Planimetry traces the CFD jet at this level, so measuring the VCA and eliminates the circular geometric assumptions of 2D-VC width, better gradeing MR severity.^7^ Limitations are workflow complexity, low spatial resolution affecting planimetry accuracy and low temporal resolution, making acquisition of a frame at exactly mid-systole challenging.

Flow convergence is another method for assessing flow through an RO. Flow accelerates as it converges towards a narrow orifice. In a circular RO, flow modelled with CFD appears as aliasing hemispheric shells increasing in velocity as they approach the RO. The distance from the RO to the first aliasing shell is the radius of a hemisphere of known area and velocity, the PISA.^6^ The continuity equation, based on conservation of mass and PISA, then enables an estimate of the effective RO area. As with VC, PISA assumes a circular RO, introducing error. Three-dimensional transoesophageal echocardiography shows a 3D PISA shell that is not a hemisphere but resembles the orifice itself, eliminating circular geometric assumptions. Poor spatiotemporal resolution of 3D-CFD datasets limits its application, but in future semi-automation of this technique may supersede 2D-PISA calculation.^8^

Assessment after MV surgery occurs after deairing the heart and weaning from cardiopulmonary bypass (CPB). When more than mild MR occurs after valve repair, the same techniques of 3D-TOE previously described can interrogate the severity and mechanism to identify reversible causes. Three-dimensional transoesophageal echocardiography can work alongside 2D-TOE to identify the location and severity of residual MR, facilitating discussions on how to proceed.

After prosthetic valve replacement, 3D-TOE enables assessment of leaflet mobility, valve rocking and using CFD, valvular and paravalvular leak.^9^ Normal mechanical valve ‘washing jets’ alongside small clinically insignificant leaks in the sewing ring can make it difficult to identify larger jets of concern. The proportion of the valve circumference the jet occupies determines the paravalvular leak severity, expressed as a clock-face using the 3D *en face* view. Recently, the ability to render tissue and the valve as translucent has made it easier to identify the leaks and their origins. An alternative approach is to render colour flow as translucent, visualising the defect behind it. Figure 1 online video shows a paravalvular leak in 2D and 3D, illustrating how translucent tissue rendering can better assess its location and extent.

#### Mitral stenosis

Mitral stenosis (MS) is seldom a repairable lesion and often requires valve replacement. However, in rheumatic MV disease, there are mixed stenotic and regurgitant components which may be amenable to repair in a subset of patients. In this setting, 3D-TOE may better identify commissural fusion, and regurgitant lesions which can undergo repair more accurately than 2D-TOE.

The grading of MS severity uses symptoms, estimated MV area (MVA), transvalvular flow, and additional upstream parameters.^6^^,^^7^ Mitral valve area measurements are often subject to inaccuracy caused by changes in left atrial and left ventricular compliance. Tracing the inner edge of the MV orifice during mid-diastole from the transthoracic parasternal short-axis (SAX) view is the gold standard for determining the anatomic MVA. Replicating this with 2D-TOE is difficult because of poor plane alignment. The MPR analysis of a stenotic MV 3D-TOE dataset can position a cross-sectional plane across the leaflet tips in a well-aligned SAX view, enabling MVA planimetry. Limitations of this technique are again achieving adequate spatiotemporal resolution to confidently trace the orifice.

### Aortic root

**Fig 2:**
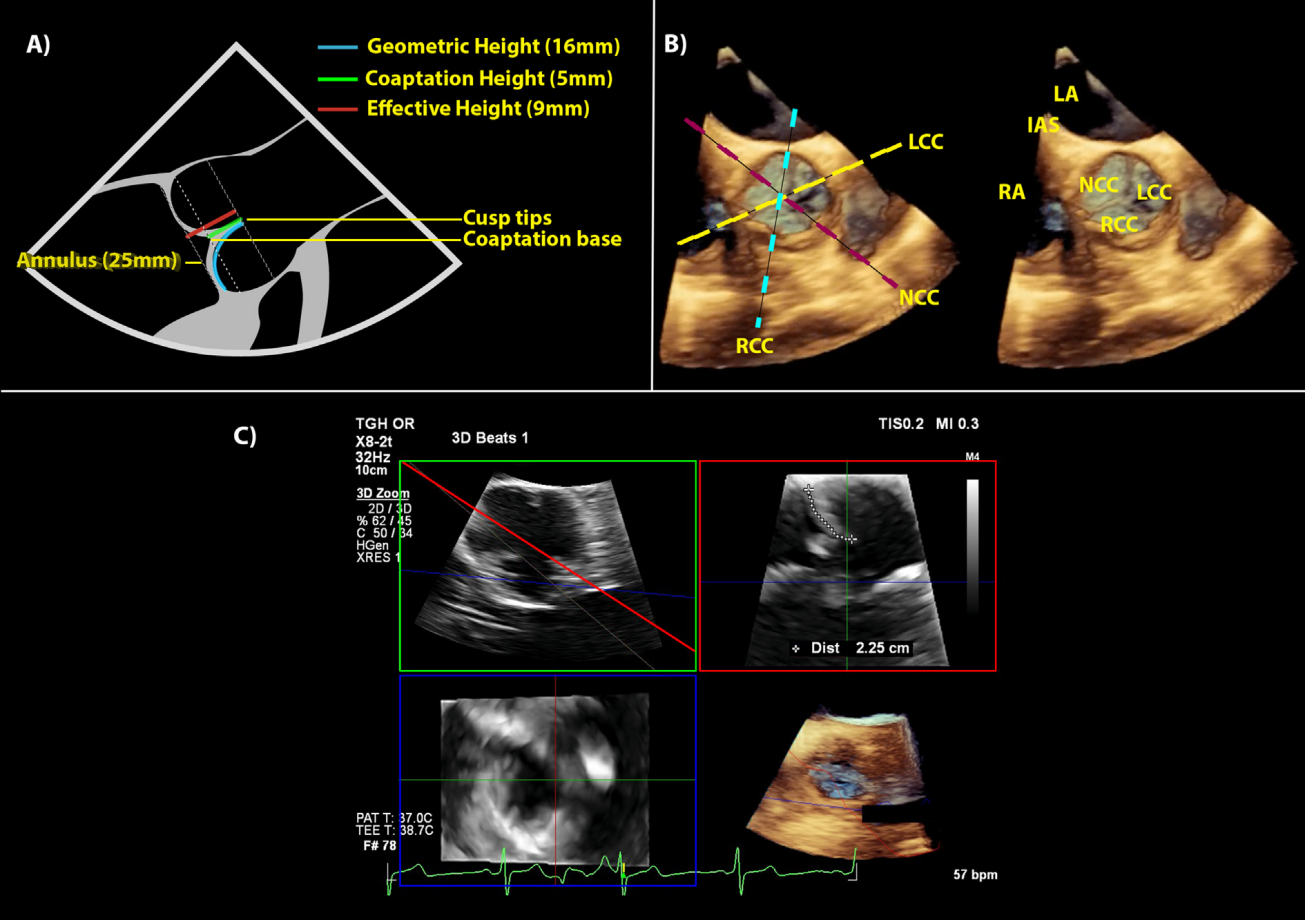
Three-dimensional transoesophageal echocardiography views of the AoV. (A) This is a diagram of the AoV and aortic root in the long axis. The geometric height, coaptation height and effective height are all measurements highlighted by coloured lines with average normal values.^12^^,^^23^ (B) This shows a 3D short axis view of the AoV looking from the aorta, the recommended orientation as per ASE guidelines. Anatomic structures are labelled on the right. Three dotted lines show planes through the centre of each cusp body and the opposing commissure in diastole. These planes guide the measurements discussed in part A. (C) This shows the measurement of the geometric height of the NCC using MPR. The AoV appears in diastole in short axis in the green box, with the red plane then aligned across the NCC body and opposing commisure. The red box shows a tracing of the geometric height of the NCC. The blue plane/box is not used. AoV, aortic valve; IAS, intra-atrial septum; LA, left atrium; LCC, left coronary cusp; LVOT, left ventricular outflow tract; MPR, multi-plane reconstruction; NCC, non-coronary cusp; RA, right atrium; RCC, right coronary cusp.

**Figure S5:**
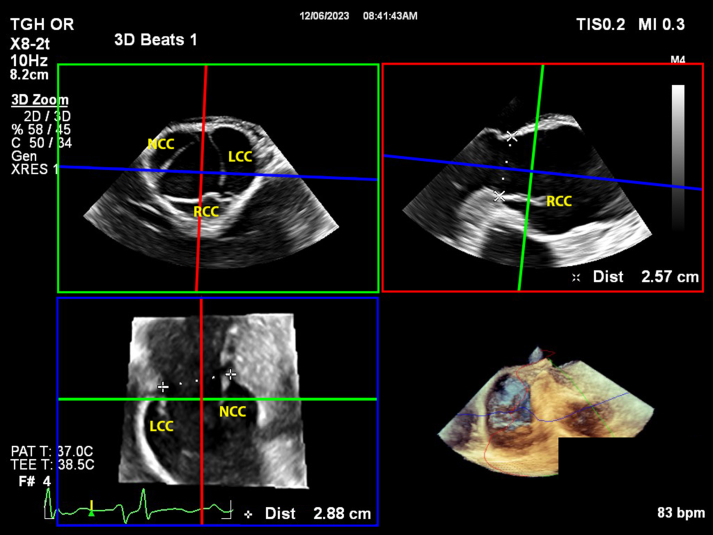
Measuring aortic root dimensions using MPR. In this aneurysmal aortic root, MPR is used to align two perpendicular planes accurately across the AoV annulus in mid-systole. The green box shows a short axis view of the AoV, which is used to align the red plane across the LCC/NCC commissure and through the mid body of the RCC. The red box displays this plane, which is a correctly aligned long-axis view of the aortic root, allowing accurate measurement of the aortic annulus in this orientation. The blue plane aligns through the NCC and LCC bodies, which is a non-standard orientation but can assess distortion of the root caused by an aneurysm. Note that the annular dimensions of 2.88 cm and 2.57 cm are markedly different. A similar assessment of the root at the level of the sinuses of Valsalva and sinotubular junction is possible. Abbreviations: AoV, aortic valve; LCC, left coronary cusp; MPR, multi-plane reconstruction; NCC, non-coronary cusp; RCC, right coronary cusp.

The aortic valve (AoV) sits in the centre of the aortic root complex which begins at the left ventricular outflow tract (LVOT) and ends at the sinotubular junction of the ascending aorta. The AoV comprises cusps and inter-leaflet triangles suspended in the sinuses of Valsalva. Thin cusps and thick calcified tissue challenges 3D-TOE imaging of the valve without artefactual ‘holes’ in the valve from tissue dropout.^2^ Despite this, some patients produce excellent 3D images. Multi-planar reconstruction offers accurate aligned measurements of the interventricular septum, LVOT, aortic annulus, sinuses and ascending aorta, which are important in many procedures and for estimating cardiac output (Fig. 2 online video). In situations of aortic root distortion from dilation or aneurysmal pathology, such as an aneurysmal sinus of Valsalva, MPR can display a plane through each sinus, assessing its size, asymmetry and suitability for repair, and the AoV morphology (Supplementary Fig. S5).

Supplementary video related to this article can be found at https://doi.org/10.1016/j.bjae.2024.04.008

The following is/are the supplementary data related to this article:

Video S2 This video focuses on the aortic valve.3 Video S2 - aortic valve

#### Aortic regurgitation

A repair based functional classification by El-Khoury describes the mechanism of aortic regurgitation (AR). Aortic regurgitation may involve root pathology, valve pathology, or a combination.^10^ Treatment of a dilated aortic root and ascending aorta, with or without AR, often uses valve replacement as part of a Bentall procedure. However, increasingly the native valve is retained in selected patients, offering improved valvular durability and the avoidance of anticoagulation.^11^ The AoV may require repair, and as with MV repair, TOE aims to confirm and refine understanding of the known pathology and any associated lesions, assess left ventricular function, and identify factors favouring repair or replacement. Failed repairs are more likely with small AoV cusps and a dilated aortic root.^12^ Multi-planar reconstruction planes derived from an aortic root 3D dataset provide precise measurements that 2D-TOE cannot achieve, in particular the ability to measure the curved distance along each individual cusp, known as the geometric height (Fig. 2).^13^ In addition, each cusp may be assessed for partial or total prolapse which is difficult in 2D alone for cusps other than the right coronary cusp, which is the only cusp that reliably aligns in a long axis view. Bicuspid valves with variable coaptation lines can also be hard to view in 2D, but may be repairable. Overall 3D-TOE can better assess aortic root pathology and aid the surgeon with AoV-sparing surgical procedures or isolated AoV repair.^14^

Analogous to MR, qualitative, semi-quantitative and quantitative methods can assess AR jets, each with limitations.^7^ 3D-TOE improves accuracy compared to 2D-TOE, specifically for measuring the VCA.

#### Aortic stenosis

**Figure S6:**
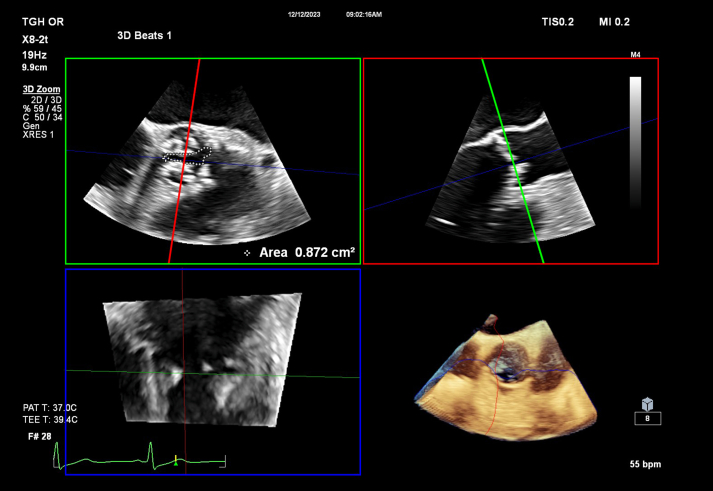
Measuring aortic valve area using MPR and planimetry. The red box shows an MPR-derived imaging plane similar to a 2D AoV long-axis view. Following identification of the leaflet tips in mid-systole, perpendicular alignment of the green imaging plane in the red box occurs at the AoV leaflet tips. The valve orifice appears in the green box and traced using planimetry (see white dotted line). The blue box/plane is not used here. Abbreviations: AoV, aortic valve; MPR, multiplane reconstruction.

Grading aortic stenosis severity uses spectral Doppler indices and estimates of the AoV area (AVA) rather than direct 2D-planimetry of the anatomic AVA. This is because direct 2D-planimetry can overestimate the AVA if the imaging plane aligns obliquely below the valve tips. Three-dimensional transoesophageal echocardiography with MPR allows precise placement of a cross-sectional plane at the leaflet tips, allowing accurate AVA planimetry (Supplementary Fig. S6).

Estimating AVA using the continuity method compares flow through a known cross-sectional area, the LVOT, with flow through an unknown area, the AVA. Left ventricular outflow tract diameter measurement by 2D-TOE is subject to error because of the geometric assumption that it is circular when it is often oval. Three-dimensional transoesophageal echocardiography reduces this error by measuring the LVOT area from MPR using planimetry. Time and complexity are again limitations.^15^

### Tricuspid valve

**Fig 3:**
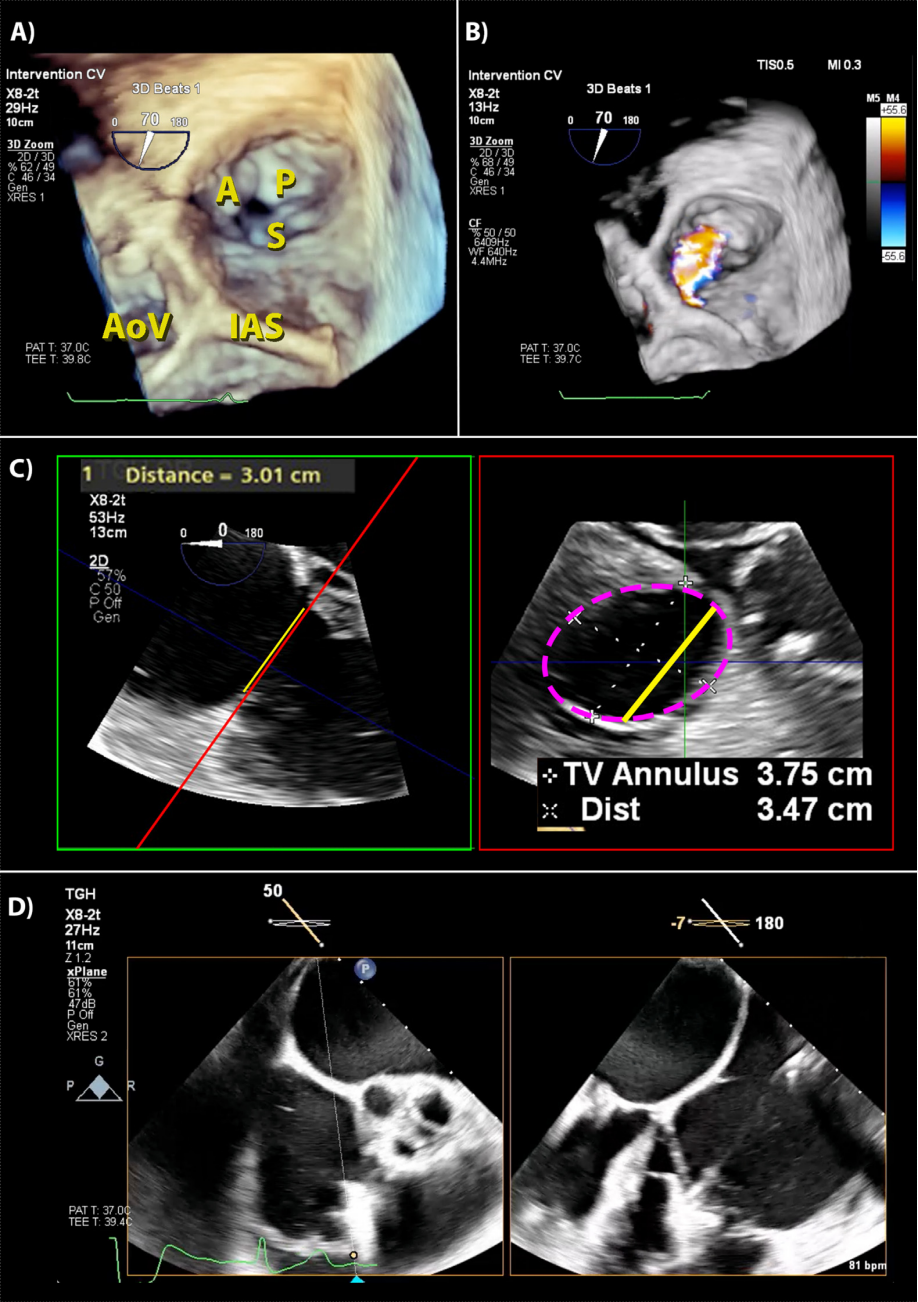
Three-dimensional transoesophageal echocardiography views of the TV. (A) This is a 3D image of the TV in diastole in a short axis orientation from the RA perspective as per the ASE guidelines. The labels of important anatomic structures are in yellow. (B) Shows part (A) (as grey scale) but with CFD superimposed showing a TR jet. (C) This shows MPR of the TV annulus. The green box is an MPR-derived 2D plane resembling the standard mid-oesophageal RV-focussed view used to measure TV annulus, as shown by the yellow line and measurement. Placing the red MPR plane across shows the valve and the annulus in the red box, with the annulus highlighted by the pink dotted line. Note these measurements are significantly different, as the 2D plane may not capture the annulus at its widest point. The yellow plane is shown in the MPR reconstruction, illustrating why 2D-TOE measurements may under-estimate annular diameter when they are not aligned at the widest point. Note also the annulus is oval and may dilate asymmetrically (see main text). (D) This shows bi-plane imaging to guide a catheter mounted clip across the TV. Note the two planes are not perpendicular (as is default) but have been adjusted to optimise the view. The left pane uses an imaging plane at 50°, capturing the valve in the standard 2D-TOE RV-inflow-outflow view. The thin white line ending in a blue arrow marks the point where the non-perpendicular plane in the right pane intersects with it. A, anterior leaflet; AoV, aortic valve; CFD, colour flow Doppler; IAS, intra-atrial septum; MPR, multi-plane reconstruction; P, posterior leaflet; RA, right atrium; RV, right ventricle; S, septal leaflet; TR, tricuspid regurgitation; TV, tricuspid valve.

Tricuspid valve (TV) pathology is mostly secondary to pressure overload from left-sided heart disease, but primary pathologies such as endocarditis may occur. Identification of the individual TV leaflets is challenging using 2D-TOE as many views show only two leaflets and transgastric basal views are difficult to obtain. Three-dimensional transoesophageal echocardiography can display all three leaflets at once, but because of its thin leaflets and distance from the probe, adequate display of all three leaflets is possible only 65% of the time (Fig. 3A, Fig. 3 online video). Biplane imaging is also useful for identifying the different leaflets. Three-dimensional transoesophageal echocardiography can quantify tricuspid regurgitation (TR) severity (Fig. 3B, Fig. 3 online video), but limitations in spatiotemporal resolution make measuring VCA challenging.

Supplementary video related to this article can be found at https://doi.org/10.1016/j.bjae.2024.04.008

The following is/are the supplementary data related to this article:

Video S3 This video focuses on assessment of the tricuspid valve.4 Video S3 - tricuspid valve

Tricuspid annular dilatation with or without TR is a common finding in patients undergoing MV surgery and is associated with worse outcomes. Tricuspid regurgitation is problematic to quantify under anaesthesia because of load-dependent changes. Typically, TV annular dilatation correlates with TR severity, and so, in the perioperative setting, measuring the annulus diameter may be a more sensitive marker for TV dysfunction. In functional TR, the annulus dilates along the anterolateral region, which 2D-TOE does not image well using standard views. Three-dimensional transoesophageal echocardiography more accurately represents annular dimensions than 2D, with measurements using MPR (Fig. 3C) correlating better in awake patients under normal loading conditions than direct measurements by the surgeon.^16^ This can help advise clinical decisions on whether to undertake TV intervention during MV surgery.

### Pulmonary valve

**Figure S7:**
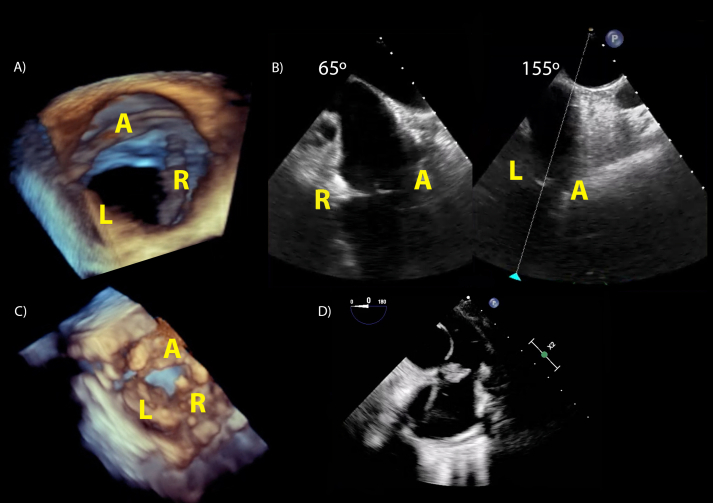
3D-TOE of the PV. (A) This shows a 3D rendered view of the PV from the MPA perspective with labelled leaflets. (B) Biplane imaging at 65° and 155° shows the PV from the upper oesophageal view. (C) This is a 3D image of a stenotic PV from the same vantage point as part A. (D) This is the 2D mid-oesophageal aorta short axis view of the same stenotic PV in part C. Abbreviations: A, anterior leaflet of PV; L, left leaflet of PV; MPA, main pulmonary artery; PV, pulmonary valve; R right leaflet of PV.

Like the TV, the pulmonary valve (PV) is difficult to image with 3D-TOE because of its remoteness from the TOE probe. However, imaging is possible and can support perioperative decision-making when searching for lesions such as endocarditis, measuring valve area for procedures such as the Ross procedure and for aligning spectral Doppler when assessing flow dynamics (Supplementary Fig. S7).

## Ventricular size and function

Assessment of left and right ventricular (RV) function is crucial during the perioperative period. It guides haemodynamic manipulation, ventilation, weaning from CPB, and identifies regional wall motion abnormalities (RWMA) present after coronary artery bypass grafting (CABG) or from iatrogenic injury to the coronary arteries. The assessment of systolic cardiac function can be qualitative or quantitative, with the latter being preferred to reduce inter-observer variability and monitor trends. In the operating theatre, haemodynamic conditions can vary significantly, requiring the monitoring of more load-independent metrics. These values, often difficult to obtain by 2D-TOE, may be acquired more easily using 3D-TOE.

### Chamber quantification

Static measurements of cardiac chamber size guides understanding of the chronicity of heart disease and assessment of global systolic function. Ejection fraction (EF) is the percentage of blood ejected from the ventricle during systole and is a global estimate of left ventricular function.^17^ Accurate measurement of EF requires perpendicularly aligned, non-foreshortened imaging planes. Foreshortening is an off-axis oblique imaging plane that shows an incomplete ventricular cavity. Foreshortening underestimates end-diastolic and end-systolic volumes, and consequently EF, and overestimates ventricular wall thickness, causing inaccurate RWMA assessment.

In assessment of the LV, biplane imaging significantly improves the reliability of chamber quantification. It reduces or eliminates foreshortening of the primary imaging plane by aligning the plane axis through the ventricular apex in simultaneous perpendicular views. Conventional measurements of left ventricular volume and EF by Simpson's biplane can occur in both planes across the same cardiac cycle. However, even when aligned, these techniques still rely on geometric assumptions about the ventricular shape and may not account for RWMA and structural changes, such as aneurysmal segments.

**Fig 4:**
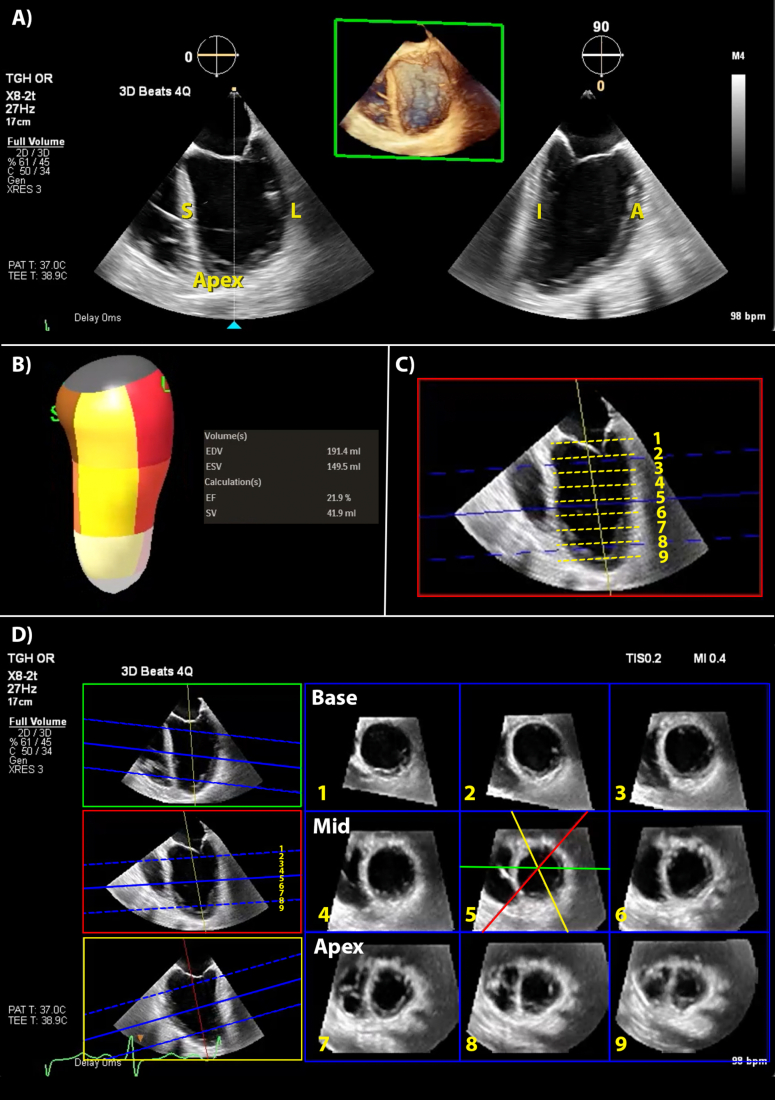
Three-dimensional transoesophageal echocardiography views of the left ventricle. (A) This is a full volume acquisition of the LV using multi-beat acquisition over 4-beats at a frame rate of 27 Hz. (B) This shows a 3D volumetric model of the LV, from tracking of the endocardial border across the cardiac cycle. Myocardial segments are colour coded. Calculation of EF without geometric assumptions occurs from this dataset. (C) Reconstruction of the left ventricular full volume acquisition can show multiple short axis planes across the ventricle. This aids in identification of RWMA. The planes, numbered 1–9, correspond to those seen in the machine display shown in part (D). (D) Multiple short-axis slices of the LV reconstructed from the full volume dataset. Three MPR planes seen in green, red and yellow planes on the left are aligned across the left ventricular cavity. Aligning the blue dotted lines between the base and apex delineates the position of the first and last short axis slices seen on the right. The addition of numbers aids understanding when viewed with part (C). A, anterior wall of left ventricle; EDV, end-diastolic volume; EF, ejection fraction; ESV, end-systolic volume; I, inferior wall of left ventricle; L, lateral wall of left ventricle; LV, left ventricle; RWMA, regional wall motion abnormalities S, intraventricular septum; SV, stroke volume.

Full volume 3D datasets of the LV at an adequate temporal resolution (>20 vols s^−1^) avoid foreshortened 2D-TOE views and eliminate the reliance on geometric assumptions (Fig. 4A, Fig. 4 online video). Semi-automated analytic software can model both the chamber and the myocardium in 3D over the cardiac cycle (Fig. 4B). The software tracks the endocardial border frame by frame, even with abnormal geometry, yielding reliable systolic and diastolic volumes and LVEF calculations. Multi-planar reconstruction of the LV can occur at multiple levels in simultaneous short axis slices, enabling qualitative assessment for RWMA (Fig. 4C).

Supplementary video related to this article can be found at https://doi.org/10.1016/j.bjae.2024.04.008

The following is/are the supplementary data related to this article:

Video S4 This video focuses on the left ventricle.5 Video S4

**Figure S8:**
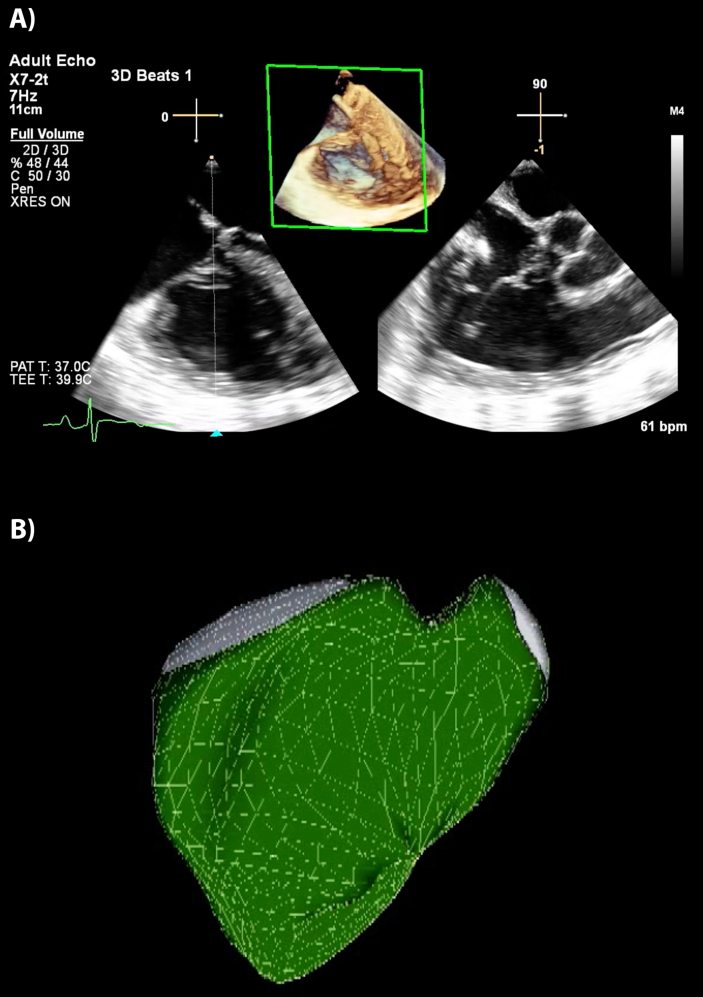
3D-TOE acquisition of the RV. (A) This shows a single beat 3D full volume acquisition of the RV, obtained from an RV focussed mid-oesophageal 4 chamber view. The volume dimensions contain the entire RV, including the RV outflow tract. (B) This is a dynamic wire-frame model of the RV endocardial border using offline processing by TomTec (Munich, Germany) software. The RV has a complex shape with the software automatically measuring ejection fraction and helping to identify RWMA. Abbreviations: RV, right ventricle; RWMA, regional wall motion abnormalities.

Analysis of a full volume RV 3D dataset using specialised software can generate an endocardial cast of the RV and provide an RVEF, something not possible with 2D-TOE. An adequate 3D dataset must include the entire RV at a frame rate of 20–25 vols s^−1^. Despite the complex shape of the RV, 3D-TOE directly measures the entire RV volume and EF, resulting in accurate and reproducible measurements (Supplementary Fig. S8).

Poor spatiotemporal resolution limits full volume chamber assessment by 3D-TOE. Limited spatial resolution does not clearly delineate endocardial trabeculae, which introduces inaccuracy in endocardial border tracking across the cardiac cycle. Poor temporal resolution may not capture the chamber volume at end-systole, therefore overestimating the end-systolic volume. Right ventricular modelling exacerbates these limitations because of greater trabeculation and difficulty imaging the free wall. Studies show 3D-TOE consistently underestimates left ventricular and RV volumes compared with cardiac MRI (CMR) although with similar accuracy.^17^ Major disadvantages for 3D-TOE are the need for specialised software and an adequate 3D dataset, limited reference values and load dependence.

### Regional wall motion abnormality

A 17-segment myocardial model classifies regional left ventricular pathology.^16^^,^^17^ To comprehensively review all segments with 2D-TOE requires an intricate analysis of a minimum of five 2D-TOE views. The assessment of RWMA relies on endocardial movement and wall thickening, which can be normal, hypokinetic, akinetic or dyskinetic. Tethering describes segments that move because of the surrounding dysfunctional myocardial contraction. Although 3D left ventricular model segmentation reflects regional myocardial segments, there is no direct assessment of wall motion or thickening.

Deformation imaging, also known as myocardial strain analysis, assesses myocardial function by measuring the actual change in myocardial length throughout the cardiac cycle. Strain and stain rate (strain/time) measurements using speckle tracking technology quantify the deformation. This analyses of 2D or 3D images identifies and tracks the movement of a unique ultrasound pattern, speckles, generated by a myocardial area through the cardiac cycle. Strain identifies areas of poor contractility with greater sensitivity than traditional global ejection indices and displays each segment on a ‘bullseye’ display.

When used with 2D images, the measurement of longitudinal myocardial movement gives global longitudinal strain. Offline analyses of a 3D dataset using specialised software permits speckle tracking in three dimensions, also measuring rotational and twisting heart motion.^18^ However, poor spatiotemporal resolution and significant inter-vendor variability affect standardisation of normal values, currently impeding the incorporation of strain measurements into guidelines.

## Cardiac masses

When presented with a cardiac mass during echocardiography, key attributes to identify are size, shape, mobility, relationship with surrounding structures and potential for resection. Two-dimensional transoesophageal echocardiography alongside spectral Doppler often provides excellent insight, but true anatomic visualisation in 3D-TOE shows the structure more intuitively and allows precise localisation and measurement of size (Fig. 1 online video).

## Congenital heart disease

Patients with congenital heart disease often have complex cardiac anatomy, often further altered by prior interventions. Multidisciplinary team discussion and review of multi-modal imaging is extremely helpful when planning and performing interventions in this group.

Standard 2D-TOE views are designed to review common anatomy. In congenital patients, visualising anatomy may be challenging and require non-standard views that are complex to interpret or communicate. Three-dimensional transoesophageal echocardiography may better capture and display structures such as ventricular septal defects, baffles and conduits, and anomalous pulmonary venous drainage, aiding peri-procedural decision-making.

Exporting full 3D-TOE datasets or CT scans to 3D graphics programs allows 3D printing of bespoke heart models.^19^ These models may be of full chambers, blood volumes or vessels. Their primary use is for planning of complex cardiac surgeries, such as congenital surgeries and interventional procedures, and in education. Model printing can use materials that mimic tissue, allowing operators to practise interventions. Durable plastic models can be an education tool for students and patients. Our institution has invested heavily in using 3D printing for these purposes with very positive feedback.

## Interventional cardiology

Advances in technology have spawned procedures that use a minimally invasive percutaneous transcatheter approach to performing structural heart interventions. These procedures involve deployment of devices to occlude holes and orifices, balloons and stents to dilate obstructions, and valves and clips to improve valvular function. Echocardiography is the cornerstone of successful procedures, facilitating pre-procedural screening, intra-procedural guidance, and post-procedure assessment.^3^ It is possible, and often desirable, to combine different imaging technologies simultaneously during a procedure. An example is using live 2D and 3D-TOE combined with fluoroscopic imaging to identify and close a paravalvular leak (Fig. 2 online video). Typically, conventional 2D planes identify the diseased structure, bi-plane imaging presents two views on a single display, before MPR and 3D rendered views help to align and show device deployment in real time.

### Trans-septal puncture

Trans-septal puncture is a common technique in many transcatheter procedures. Incorrect puncture location and the inadvertent injury to structures surrounding the intra-atrial septum (IAS) are potentially catastrophic, making catheter guidance crucial with 2D and 3D-TOE alongside fluoroscopy.^20^ Three-dimensional transoesophageal echocardiography with biplane imaging can identify needle tenting on the IAS to optimise catheter position and angle before puncture.

### Atrial septal defect

**Figure S9:**
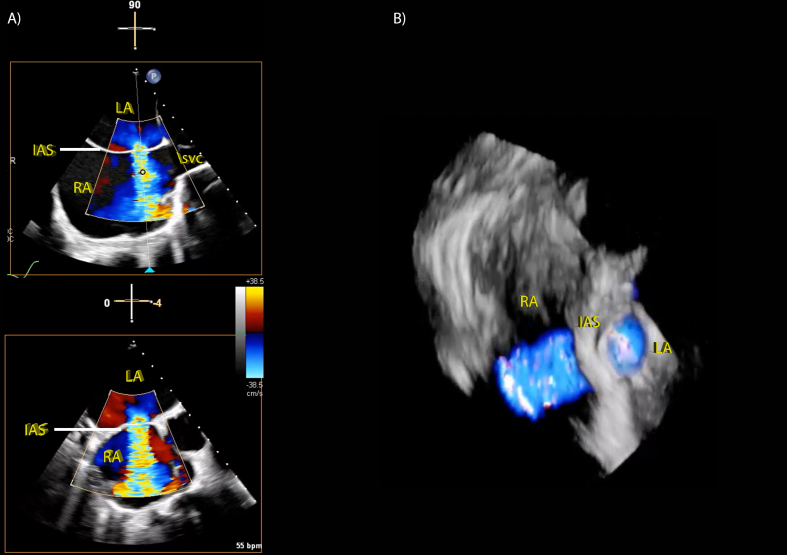
Imaging IAS with 3D-TOE. (A) This is a bi-plane image of the IAS with CFD overlay highlighting an iatrogenic ASD with left-to-right flow. The top pane shows a standard mid-oesophageal bicaval view at 90° and the bottom is at 0°. (B) This shows the same ASD in 3D from the RA perspective towards the TV, with labelling of key anatomic structures. Abbreviations: ASD, atrial septal defect; CFD, colour flow Doppler; IAS, intra-atrial septum; LA, left atrium; RA, right atrium; SVC, superior vena cava; TV, tricuspid valve.

Three-dimensional transoesophageal echocardiography shows the IAS with excellent spatial resolution, enhancing characterisation of defects, such as atrial septal defect (ASD) and patent foramen ovale (Supplementary Fig. S9). Three-dimensional transoesophageal echocardiography images allow assessment for percutaneous *vs* surgical closure of these defects, and peri-procedural support for deployment of percutaneous devices.^21^ Three-dimensional colour flow Doppler also helps locate residual leaks after deployment of percutaneous ASD occluders, particularly in a fenestrated septum.

### Percutaneous mitral interventions

The MitraClip (Abbott, Menlo Park, CA, USA) and PASCAL (Edwards Lifesciences, Irvine, CA, USA) devices are percutaneous clips that deploy across the MV, replicating an edge-to-edge surgical repair by approximating the leaflets to treat primary or secondary MR. Compared with 2D, 3D-TOE better locates the origin of multiple or eccentric MR jets. Biplane imaging optimises the trans-septal puncture, which varies in position according to leaflet pathology. Multi-planar reconstruction, 3D-Live and 3D-CFD help the operator optimise clip alignment, positioning and deployment (Fig. 1 online video). After deployment, it is possible to measure the MVA of both orifices precisely using MPR to ensure adequate transmitral flow.^22^

### Percutaneous tricuspid interventions

As with the MV, it is possible to deploy clips between the leaflets of the TV. The clips, which improve coaptation and reduce TR, are most often placed on the free margins of the leaflets anywhere between the antero-septal and the postero-septal commissures. Three-dimensional transoesophageal echocardiography is mandatory for the procedure, including multiplane imaging, live MPR and 3D rendered views (Fig. 3E). Transoesophageal echocardiography identifies the origin of the regurgitant jet to guide clip placement, avoid pacemaker leads and assess for stenosis after the clip deployment.

### Left atrial appendage

**Figure S10:**
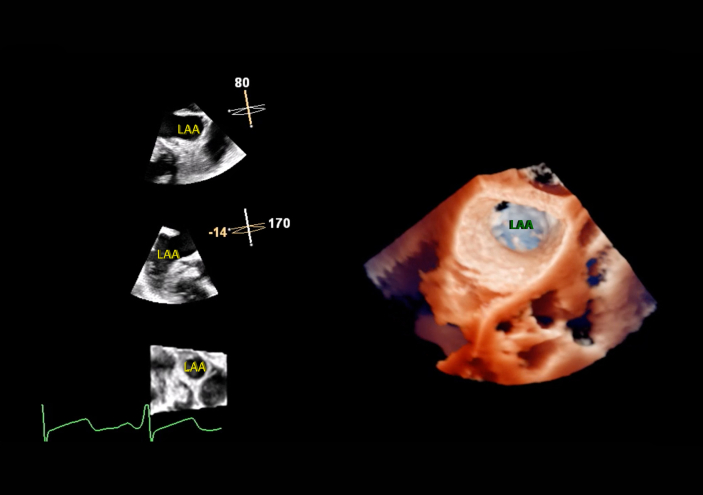
3D-TOE interrogation of LAA. A short-axis 3D image of the LAA looking down into it from the LA with tissue rendering and ray tracing technology alongside multi-plane reconstruction showing perpendicular 2D views (80° and 170°) and a short axis view of the LAA orifice on the left. Abbreviations: LA, left atrium; LAA, left atrial appendage.

The left atrial appendage (LAA) is a blind-ending pouch in the LA and a common location of thrombus formation in patients with atrial fibrillation (AF) or low cardiac output states. It is routine to interrogate the LAA during perioperative TOE to exclude clots. Three-dimensional transoesophageal echocardiography, using multi-plane imaging (Supplementary Fig. S10, Supplementary Fig. 1 online video) or 3D-CFD, can augment 2D and spectral Doppler and should be part of the assessment.^5^ Left atrial appendage closure is an alternative to long-term anticoagulation in patients with AF who are at a high risk of stroke and intolerant of anticoagulants. Percutaneous device closure is an option for those at-risk patients not undergoing cardiac surgery. Procedural steps and echocardiographic focus are similar for all percutaneous LAA occlusion devices and include central venous access via a femoral vein, trans-septal puncture to access the LA, followed by manipulations specific to the deployment of different occluders.^3^ Three-dimensional transoesophageal echocardiography is invaluable for measuring the LAA dimensions and to guide device deployment.

### Transcatheter aortic valve insertion

Transcatheter aortic valve insertion (TAVI) procedures have become increasingly common in recent years as an alternative to open AoV surgery. Preoperative measurement of the native AoV is crucial to selecting an appropriate prosthetic valve. Computed tomography angiography is the gold standard for these measurements, but 3D-TOE derived data correlate well and may benefit patients with impaired renal function to reduce contrast exposure.^23^ As most TAVI procedures occur under conscious sedation, transthoracic echocardiography has replaced TOE for immediate assessment of valve function and paravalvular leak.

## Summary

Three-dimensional transoesophageal echocardiography is an excellent tool that is still developing in the perioperative environment. Most routine use is for biplane 2D imaging and 3D *en face* views of the MV, working as a complementary technology alongside established M-mode, 2D-TOE and spectral Doppler-based protocols. Time, experience, limited spatiotemporal resolution, poor software interfaces and a lack of standardised data for perioperative 3D-TOE are all limitations. Many of these issues have improved in the last decade with better technology, artificial intelligence and 3D display options. This remains an exciting and developing area of perioperative imaging.

## Declaration of interests

The authors declare that they have no conflicts of interest.

## MCQs

The associated MCQs (to support CME/CPD activity) will be accessible at www.bjaed.org/cme/home by subscribers to *BJA Education*.
