# Supplementary material for: Perioperative 3D transoesophageal echocardiography. Part 2: clinical applications
Source: BJA Educ. 2024 Jun 17;24(8):277–87. doi: 10.1016/j.bjae.2024.04.008 (PMC11293589; doi:10.1016/j.bjae.2024.04.008)
Supplement: Multimedia component 1 [file mmc1.docx]

# Supp Fig S1

**MV *en-face* view.** This summarises the directions to obtain the surgical en face view of the MV. Specific steps vary between different platforms but are broadly similar. Abbreviations: AoV, aortic valve; CFD, colour flow Doppler; LA, left atrium; MV, mitral valve.

# Supp Fig S2

**Parametric MV models.** (A) This is a dynamic Siemens (Erlangen, Germany) model of the MV, which overlies the 3D full volume dataset and integrates CFD across the entire cardiac cycle. (B) This is a dynamic MV model during systole analysed using TomTec (Munich, Germany) software. (C) This shows a static mid-systole Philips (Amsterdam, Netherlands) MV model, identifying scallops on both leaflets and the coaptation line. Each software automatically derives and displays indices for these models. Abbreviations: CFD, colour flow Doppler; MV, mitral valve.

# Supp Fig S3

**Carpentier classification of MR.** Category I is MR with normal leaflet motion, typically from clefts or perforation. The example shows a colour jet that is not originating from the coaptation line. The 3D en-face view shows a perforation in the anterior MV leaflet near the annulus (red circle). Category II is MR with excessive leaflet motion. The example shows posterior leaflet prolapse in the 2D view, although it is unclear which segment is involved. The 3D en-face view highlights a flail P2 segment. Category IIIa is leaflet restriction in systole and diastole, often caused by calcific or rheumatic valves. The 2D image shows a thickened rheumatic valve, also seen in the 3D en-face view in diastole. Category IIIb is restriction in systole only from left ventricular ischaemia, leading to annular dilatation or RWMA that tether the chordae tendinae. Abbreviations: MV, mitral valve; MR, mitral regurgitation; RWMA, regional wall motion abnormalities.

# Supp Fig S4

**Measuring VCA using MPR**. As described in the main text, VCA measurements can estimate MR severity. The 2D image in the green box derives from the 3D dataset and resembles a conventional 2D mid-oesophageal 4 chamber view of the MV. The red box is a 2D plane perpendicular to the green. Both planes align across the MR jet orifice. The blue plane aligns perpendicular to both the red and green planes at the level of the RO. The RO appears in short axis in the blue box and traced using planimetry to yield the VCA of 4.10cm^2^ (see white dotted line in blue box). Note that in this example, there is a second MR jet seen in both the red and blue panels that is not being measured. 3D-TOE can help in isolation and measurement of multiple jets like this, which can be challenging with 2D imaging alone. Abbreviations: AMVL, anterior mitral valve leaflet; MPR, multiplane reconstruction; MR, mitral regurgitation; MV, mitral valve; PMVL, posterior mitral valve leaflet; RO, regurgitant orifice; VCA, vena contracta area.

# Supp Fig S5

**Measuring aortic root using MPR.** In this aneurysmal aortic root, MPR is used to align two perpendicular planes accurately across the AoV annulus in mid-systole. The green box shows a short axis view of the AoV, which is used to align the red plane across the LCC/NCC commissure and through the mid body of the RCC. The red box shows this plane, which is a correctly aligned long-axis view of the aortic root, allowing accurate measurement of the aortic annulus in this orientation. The blue plane aligns through the NCC and LCC bodies, which is a non-standard orientation but can assess distortion of the root because of its aneurysm. Note that the annular dimensions of 2.88cm and 2.57cm are markedly different. A similar assessment of the root at the level of the sinuses of Valsalva and sinotubular junction is possible. Abbreviations: AoV, aortic valve; LCC, left coronary cusp; MPR, multi-plane reconstruction; NCC, non-coronary cusp; RCC, right coronary cusp.

# Supp Fig S6

**Measuring aortic valve area using MPR and planimetry.** The red box shows an MPR-derived imaging plane similar to a 2D AoV long-axis view. Following identification of the leaflet tips in mid-systole, perpendicular alignment of the green imaging plane in the red box occurs at the AoV leaflet tips. The valve orifice appears in the green box and traced using planimetry (see white dotted line). The blue plane is not used here. Abbreviations: AoV, aortic valve; MPR, multiplane reconstruction.

# Supp Fig S7

**3D-TOE of the PV.** (A) This shows a 3D rendered view of the PV from the MPA perspective with labelled leaflets. (B) Biplane imaging at 65° and 155° shows the PV from the upper oesophageal view. (C) This is a 3D image of a stenotic PV from the same vantage point as part A. (D) This is the 2D mid-oesophageal aorta short axis view of the same stenotic PV in part C. Abbreviations: A, anterior leaflet of PV; L, left leaflet of PV; MPA, main pulmonary artery; PV, pulmonary valve; R right leaflet of PV.

# Supp Fig S8

**3D-TOE acquisition of the RV.** (A) This shows a single beat 3D full volume acquisition of the RV, obtained from a right ventricular-focussed midoesophageal 4 chamber view. The volume dimensions contain the entire RV, including the right ventricular outflow tract. (B) This is a dynamic wire-frame model of the right ventricular endocardial border using offline processing by TomTec (Munich, Germany) software. The RV has a complex shape with the software automatically measuring ejection fraction and helping to identify RWMA. Abbreviations: RV, right ventricle; RWMA, regional wall motion abnormalities.

# Supp Fig S9

**Imaging IAS with 3D-TOE.** (A) This is a bi-plane image of the IAS with CFD overlay highlighting an iatrogenic ASD with left-to-right flow. The top pane shows a standard mid-oesophageal bicaval view at 90° and the bottom is at 0°. (B) This shows the same ASD in 3D from the RA perspective towards the TV, with labelling of key anatomic structures. Abbreviations: ASD, atrial septal defect; CFD, colour flow Doppler; IAS, intra-atrial septum; LA, left atrium; RA, right atrium; SVC, superior vena cava; TV, tricuspid valve.

# Supp Fig S10

**3D-TOE interrogation of LAA.** A short-axis 3D image of the LAA looking down into it from the LA with tissue rendering and ray tracing technology alongside multi-plane reconstruction showing perpendicular 2D views (80° and 170°) and a short axis view of the LAA orifice on the left. Abbreviations: LA, left atrium; LAA, left atrial appendage.

# Supp Video A

This video focuses on the left atrial appendage.
